# Determination of Six Macrolide Antibiotics in Chicken Sample by Liquid Chromatography-Tandem Mass Spectrometry Based on Solid Phase Extraction

**DOI:** 10.1155/2019/6849457

**Published:** 2019-02-24

**Authors:** Chen Lan, Dan Yin, Zhicong Yang, Wuduo Zhao, Yanlong Chen, Wenfen Zhang, Shusheng Zhang

**Affiliations:** ^1^College of Chemistry and Molecular Engineering, Zhengzhou University, Zhengzhou, China; ^2^Center for Advanced Analysis and Computational Science, Zhengzhou University, Zhengzhou, China

## Abstract

In this paper, a simple and effective method for the determination of six macrolide antibiotics (MACs), including tylosin, tilmicosin, azithromycin, clarithromycin, roxithromycin, and kitasamycin, in the chicken sample using liquid chromatography-tandem mass spectrometry (LC-MS/MS) was developed based on a self-built porous aromatic framework- (PAF-) based solid phase sorbent. The main parameters influencing the extraction efficiency, such as sorbent amounts, type of the eluent, pH of the sample, and the eluent volume, were evaluated. Under the optimized condition, the limits of detection were from 0.2 to 0.5 *μ*g·kg^−1^. The recoveries of the method ranged from 82.1% to 101.4% with the relative standard deviations less than 11.1%. All the results demonstrated that the established method is potential for the determination of macrolide antibiotics in food safety analysis and monitoring.

## 1. Introduction

Macrolide antibiotics (MACs) are a class of lipophilic compounds and broad-spectrum antibacterial agents produced by actinomycetes or micromonospora, consisting of 12–16 carbonolactone rings [1–3]. Due to their strong antibacterial activity against pathogens such as Gram-positive bacteria and Gram-negative bacteria, MACs are widely used in the treatment of human diseases, and as well as in the prevention and control of animal diseases in animal husbandry [3–5]. Although this type of antibiotic is less toxic, inappropriate or abusive use of antibiotics in farm animals might provoke their residues in food of animal origin and cause contamination of animal-derived food. And, the accumulation of drugs in edible animal tissues could be a potential hazard to human health. Once ingested by human body and accumulated to a certain concentration, MACs and their metabolites may cause damage to the human vestibule and cochlear nerves, even to liver and kidneys, and may lead to an increase in human resistant strains [6–9]. The investigations pointed out that food consumption is the major source of human inadvertent antibiotics intake [10, 11]. In United States, European Union, China, and many other countries, maximum residue limits (MRLs) for MACs in edible animal tissues have been set up [5]. Therefore, it is of great significance to establish a rapid, sensitive, and reproducible method for the determination of macrolide antibiotics in animal-derived foods.

Recently, with the development of analytical instruments, various analytical methods have been gradually developed to determine the trace drug residues in complex matrices, including capillary electrophoresis (CE) [12–14], enzyme-linked immunosorbent assay (ELISA) [15], thin-layer chromatography (TLC) [16], voltammetric measurements [17], gas chromatography coupled to mass spectrometry (GC-MS) [18, 19], high-performance liquid chromatography (HPLC) [20–22], and liquid chromatography coupled with tandem mass spectrometry (LC-MS/MS) [2, 23–27]. Among these analytical methods, LC-MS/MS appears to be acknowledged as the most useful and authoritative methods for the quantification of MACs in complex matrices, due to its high sensitivity and good specificity [24, 26].

However, the matrix of animal-derived food samples is complex, and the impurities such as fat and protein existed in animal tissues samples, which not only affects the separation and quantitative analysis of target analytes but also may pollute the chromatographic column and analytical instruments. Therefore, proper sample preparation is important.

Until now, several sample preparation methods including solid-phase extraction (SPE) [28], liquid-liquid extraction (LLE) [29, 30], matrix solid-phase dispersion (MSPD) [9], pressurized liquid extraction (PLE) [31], and magnetic solid-phase extraction (MSPE) [5] have been used to extract macrolide antibiotics from foodstuff. And, SPE is one of the most frequent extraction and clean-up procedures in food fields, environment, and biomedical field [3, 32–34]. To date, a variety of different SPE sorbents have been developed to enrich antibiotics. For example, restricted access material has been used as SPE sorbent for adsorption and determination MACs [35], and mesoporous MCM-41 silica sorbent for simultaneous purification and enrichment of five MACs in mini-SPE [3]. And, poly (1-vinylimidazole-co-trimethylolpropane trimethacrylate) is used as a selective sorbent material for determination of MACs in mineral water and juice sample, etc. [36] .

In our laboratory, we have developed some efficient SPE sorbents including calixarene [37], ion liquids [38], metal organic framework (MOF) [39], covalent organic framework (COF) [40], and porous aromatic framework sorbents [41, 42] and have been used in the analysis of the different targets. Among these sorbents, the porous aromatic framework displayed the excellent adsorption performance for multiple analytes [41, 42].

**Figure 1:**
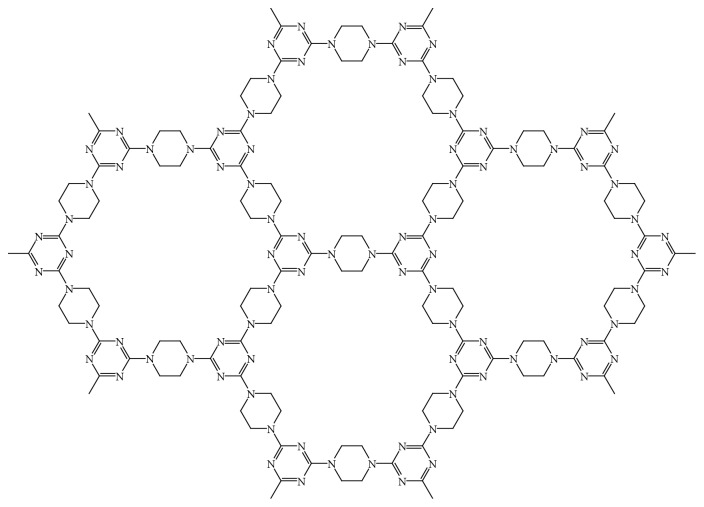
The chemical structure of PAF-6.

Porous aromatic frameworks (PAFs) possess high surface areas, high porosity, intrinsic electron rich structure, high chemical and thermal stabilities, *π*-*π* conjugated array systems, a specific hydrophobic-hydrophilic nature, and many other advantages due to the numerous existent of aromatic builder units in its structure [43, 44]. In this work, we synthesized porous aromatic framework (PAF-6) between two organic monomers, cyanuric chloride, and piperazine. The chemical structure of PAF-6 is shown in Figure 1. The aromatic rings and nitrogen atoms in the PAF-6 framework endow it with multipoint recognition sites and versatile adsorption capacity [41]. Considering its lack of toxicity and high chemical stability, PAF-6 was applied as a SPE sorbent to selectively and feasibly extract and purify six MACs from chicken samples in the present work. The LC-MS/MS method for the simultaneous separation and determination of six MACs in chicken foods was developed with high sensitivity and selectivity.

## 2. Experimental

### 2.1. Solvents and Reagents

**Figure 2:**
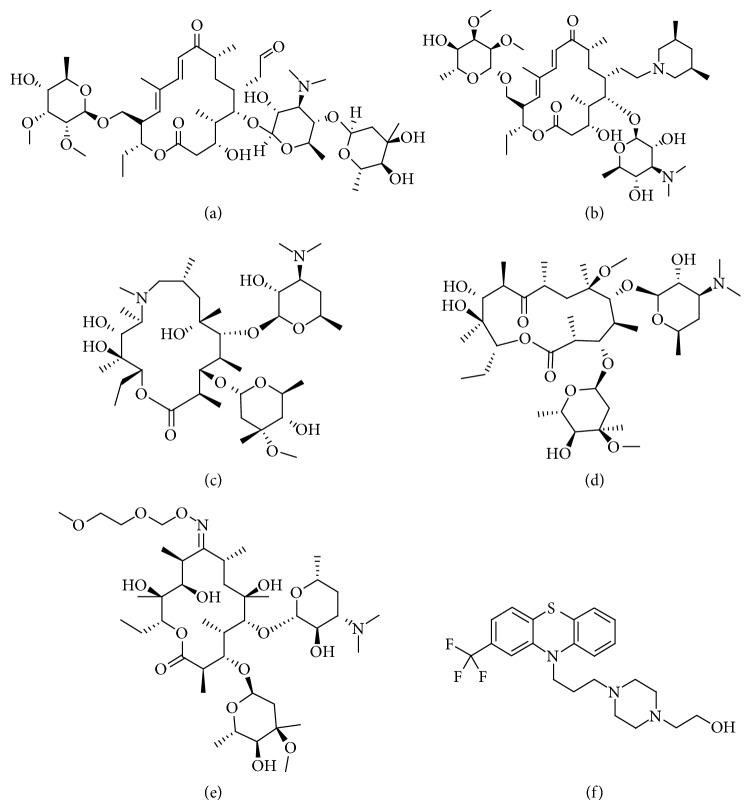
Chemical structures of the studied macrolide antibiotics. (a) Tylosin. (b) Tilmicosin. (c) Azithromycin. (d) Clarithromycin. (e) Roxithromycin. (f) Kitasamycin.

Methanol and acetonitrile of HPLC grade were obtained from Thermo Fisher Scientific (USA); HPLC grade formic acid was purchased from Amresco (USA); other reagents used in the experiment were all analytical grade. Deionized water was purified by a Milli-Q system from Millipore (Millipore, USA). The polypropylene column and 20 *μ*m PTFE sieve plates used for SPE were purchased from Dikma (Dikma, Germany). The chicken samples used in the experiment were provided by the Henan Province Bureau of animal husbandry. Tylosin, tilmicosin, azithromycin, clarithromycin, roxithromycin, and kitasamycin (purity > 98%) were purchased from the China Institute of Veterinary Drugs Control. The chemical structures of the six MACs are shown in Figure 2. The stock standard solution was prepared by dissolving tylosin, tilmicosin, azithromycin, clarithromycin, roxithromycin, and kitasamycin in methanol. The stock solution was stored at 4°C in the refrigerator. We got the working standard solutions by stepwise diluting of stock solutions with methanol/water (v/v, 20 : 80). All standard solutions were stored at 4°C prior to use. For PAF-6, the structure and synthesis have been reported in detail elsewhere [42, 45].

### 2.2. Sample Preparation and Extraction Procedure

The preparation process of chicken samples was according to the previous literature with some modification [21]. First, the chicken samples were homogenized in a homogenizer. 2.0 g of homogenized sample was weighed into a 50 mL centrifuge tube, and spiked with 200 *μ*L of 0.1 mol/L EDTA solution and 10 mL of acetonitrile/methanol (v/v, 95 : 5). After continuous vortexing and shaking for 20 min and centrifuging at 5000 r/min for 5 min, the supernatant was transferred to a 25 mL heart-shaped bottle. 10 mL of acetonitrile/water (v/v, 15 : 2) was added to the residue for repeated extraction, and the two supernatants were combined. The bottle was evaporated until the remaining solution was about 1 mL after adding 0.4 g of NaCl. Finally, the bottle was washed with 1 mL of acetonitrile and 15 mL of water, and the eluent was collected into a 50 mL centrifuge tube as a stock solution.

The pretreatment procedure of PAF-6 SPE cartridge (60 mg/3 cc): first, the cartridges were prepared by packing 60 mg of PAF-6 into the empty polypropylene SPE cartridges (3 mL). Then, 8 mL spiked sample solution or extracting solution was passed through the cartridges, which have been preconditioned with 3 mL methanol and 3 mL water at a flow rate of 4.0 mL·min^−1^. Second, the cartridges were washed with 5 mL of water at the flow rate of 1.0 mL·min^−1^. Finally, the analytes were eluted with 5 mL 5% ammoniated methanol at the flow rate of 1.0 mL·min^−1^. The collected eluent was concentrated under a gentle stream of nitrogen and then was redissolved to 1.0 mL with mobile phase, then used for the following LC-MS/MS analysis.

### 2.3. LC-MS/MS Analysis

The chromatographic separation was carried out using a Thermo Scientific Hypersil GOLD C18 column (2.6 *μ*m, 100 mm × 2.1 mm) at 35°C with an injection volume of 5 *μ*L. The flow rate was 0.3 mL·min^−1^. The mobile phases were composed of 0.10% formic acid solution as mobile phase A and methanol as mobile phase B.

A TSQ QUANTIVA (ThermoFisher, USA) triple quadrupole mass spectrometer equipped with an electron spray ionization (ESI) interface, operating in the positive-ion mode, was used. The optimum conditions of selective reaction monitoring (SRM) were carried out at the following parameters: ion spray voltage, 3500 V; auxiliary gas pressure, 5 arb. units; ion transfer tube temperature, 350°C; ion source temperature, 300°C. The values of collision energy, transitions for the SRM mode, are given in Table 1.

### 2.4. Theoretical Computations

According to the density functional theory (DFT), we investigate interactions between host and guest molecules [46]. In this paper, B3LYP/6–31 + G was used to calculate the geometry optimizations between PAF-6 and MACs. All theoretical calculations were carried out using the Gaussian 09 package.

## 3. Results and Discussion

### 3.1. Molecular Interaction Mechanism

To investigate the molecular interaction mechanism, a theoretical calculation was performed based on the DFT-B3LYP using 6-31G as the basis set [46], according to the following formula:

(1) $$\begin{array}{c} \Delta E=E_{\text{PAF}-\text{6}-\text{MACs}}-E_{\text{PAF}-6}-E_{\text{MACs}}. \end{array}$$

**Figure 3:**
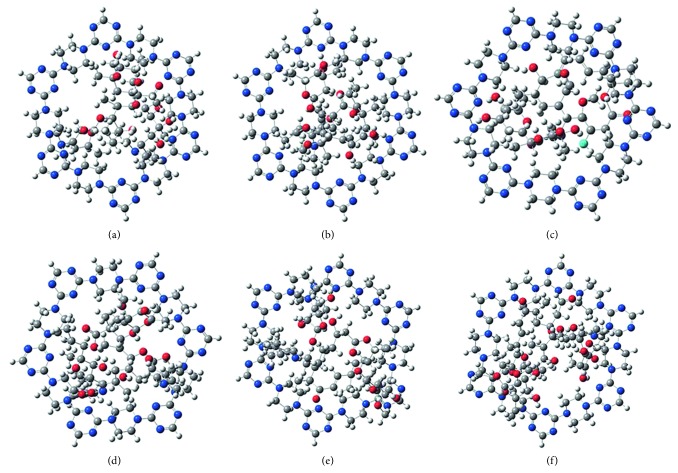
Possible structures of the PAF-6-analyte complexes: (a) PAF-azithromycin, (b) PAF-clarithromycin, (c) PAF-kitasamycin, (d) PAF-roxithromycin, (e) PAF-tilmicosin, and (f) PAF-tylosin. Carbon: gray; hydrogen: white; oxygen: red; nitrogen: blue.

The values of *E*_MACs_, *E*_PAF−6_, *E*_PAF−6−MACs_, and Δ*E* are listed in Table 2. These data showed that MACs could spontaneously adsorb onto the PAF-6 molecules. From Figure 3, we can speculate that inclusion complexation and hydrogen-bonding interaction of host-guest existed in PAF-6 and MACs.

### 3.2. LC-MS/MS Optimization

A full scan mass spectrum was obtained for each MAC and then examined to determine the precursor ion. To obtain the most selective and sensitive product ions of each MAC, a product ion scan was performed by applying an energy ramp between 10 and 50 V. The collision energy was fully optimized for the selected transitions for each MAC. The most sensitive transitions were selected for quantification, and the secondary transitions were used for confirmation. The transitions and optimal conditions are listed in Table 1.

To achieve satisfactory separations and high responses for all target MACs, the optimal separation was achieved on Thermo Scientific Hypersil GOLD C18 (2.6 *μ*m, 100 mm × 2.1 mm) columns. Acetonitrile/water and methanol/water were tested for the separation of target compounds during the method development. The results showed that, when the methanol/water was used as the mobile phase, the response of each target was higher than that of acetonitrile/water. In order to increase the ionization efficiency of the target, we changed the aqueous phase to a 0.1% formic acid solution and found that the tailing of the target peak was reduced and the response was improved. Therefore, methanol/0.1% formic acid was chosen as the mobile phase. In addition, in order to improve the resolution of six of MACs, a gradient elution method was used in this experiment. The optimal conditions are listed in Table 3.

### 3.3. Optimization of Conditions for the SPE of MACs

In this section, the main parameters affecting the extraction efficiency of MACs using PAF-6 SPE cartridges were evaluated. All the experiments were performed in triplicate.

#### 3.3.1. Effect of PAF-6 Amount

**Figure 4:**
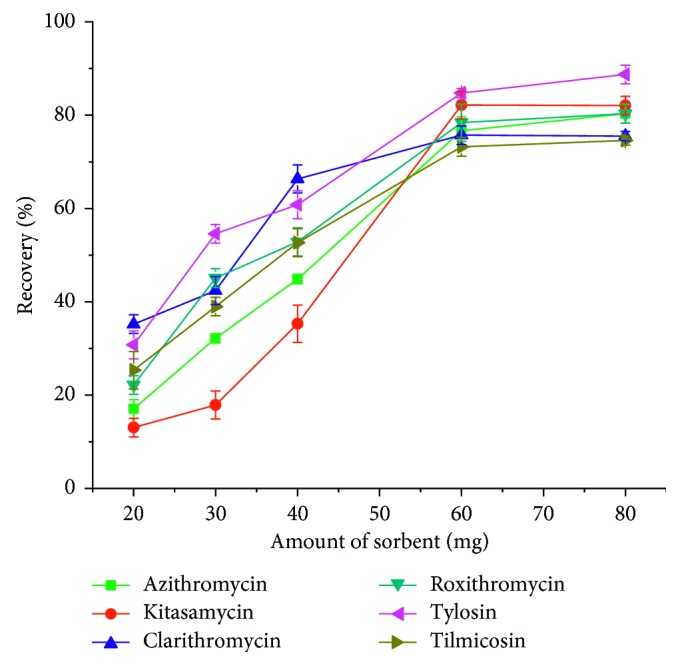
Effect of sorbent amounts on macrolide antibiotics recovery.

To achieve good recoveries of MACs, the amounts of PAF-6 were investigated with the amount ranging from 20 to 80 mg. As shown in Figure 4, it indicated that the recoveries of six target compounds went up as the amount of PAF-6 increased from 20 to 60 mg and then changed slightly from 60 to 80 mg. Thus, 60 mg amount of PAF-6 was selected as the optimum amount of the sorbent for the extraction of target MACs in the following experiments.

#### 3.3.2. Effect of Type of Elution Solvent

**Figure 5:**
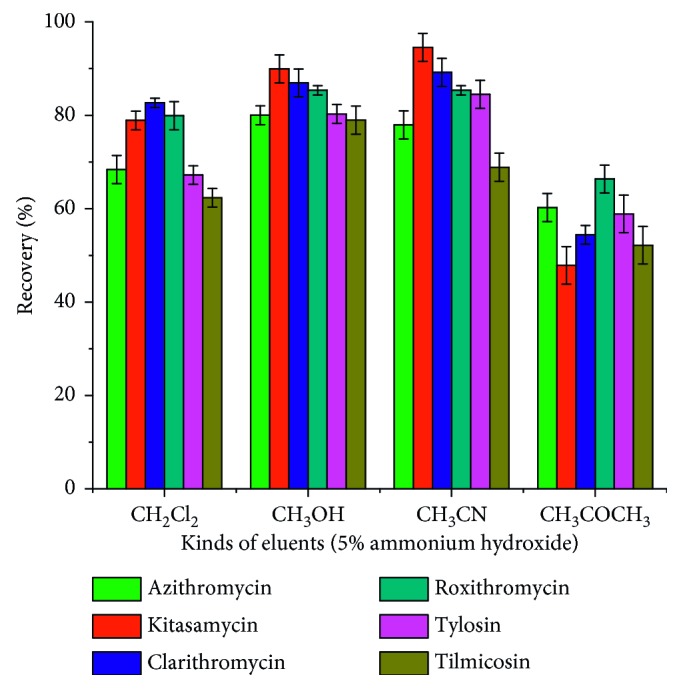
Effect of eluent type on macrolide antibiotics recovery.

**Figure 6:**
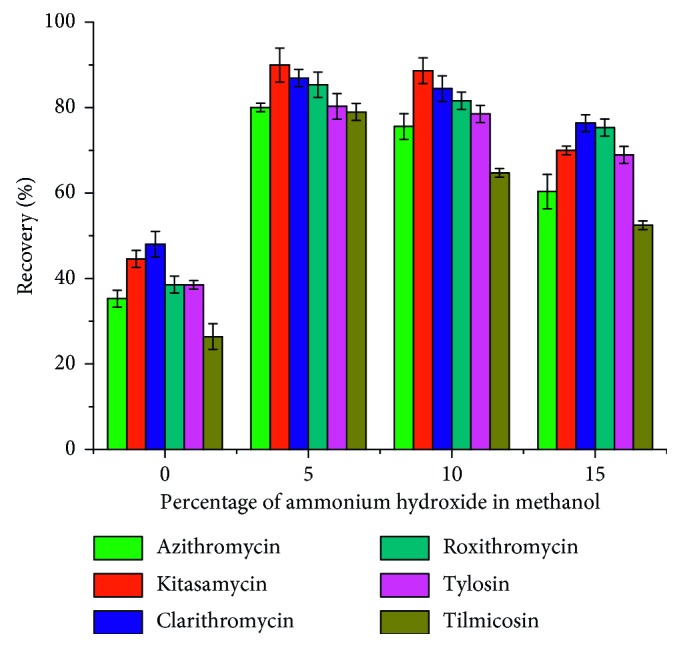
Effect of ammonia amounts on macrolide antibiotics recovery.

The eluent directly affects the desorption efficiency. In this work, methanol, acetonitrile, acetone, and dichloromethane were investigated as eluents. However, it was found that the elution power of the above four solvents on the target was weak, and the recoveries rate were lower than 50%. Considering the interaction between the target molecule and the porous material PAF-6, a small amount of ammonia hydroxide was added. It can be seen from Figure 5 that 5% aminated acetonitrile and 5% aminated methanol elute the target better than 5% ammoniated acetone and 5% aminated dichloromethane. However, the elution capacity of 5% aminated methanol and the 5% aminated acetonitrile corresponding to the target was almost equivalent. Taking into account the cost of the experiment and the safety of the experiment, a relatively inexpensive, less toxic methanol was chosen as the eluent. After that, we examined the effect of methanol with different amounts of ammonia hydroxide on the recoveries of the MACs. It can be seen from Figure 6 that when the amount of ammonia hydroxide in methanol is 5%, the recoveries of the MACs were high, so 5% ammoniated methanol was accepted.

#### 3.3.3. Effect of Volume of Elution Solvent

**Figure 7:**
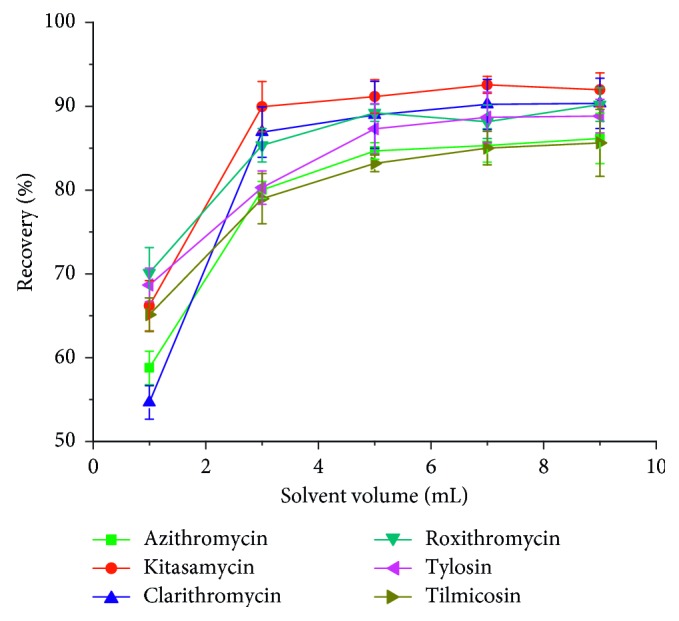
Effect of eluent volume on macrolide antibiotics recovery.

The volume of the eluent is another factor that affects the SPE recovery. As shown in Figure 7, the recoveries of MACs increased with the increase of eluent volume from 1 to 5 mL and then changed slightly from 5 mL to 9 mL. Therefore, 5 mL 5% ammoniated methanol was chosen as the volume of eluent.

#### 3.3.4. Effect of Sample Flow Rate

**Figure 8:**
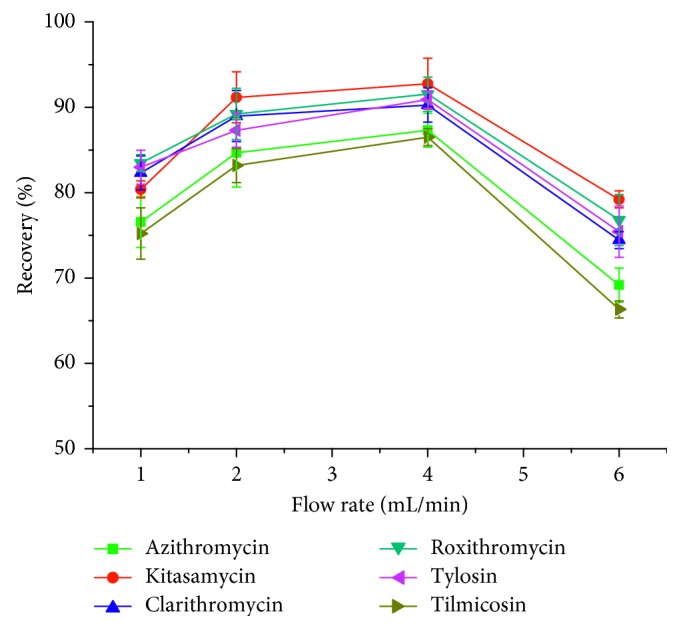
Effect of sample flow rate on macrolide antibiotics recovery.

Optimization of sample flow rate was conducted over the range of 1 to 6 mL·min^−1^. The results (Figure 8) showed that the extraction recoveries increased obviously as the flow rate increases from 1 to 4 mL·min^−1^ and then decreased, which indicated that 4 mL·min^−1^ was the optimal flow rate for further experiment.

#### 3.3.5. Effect of pH of Sample Solution

**Figure 9:**
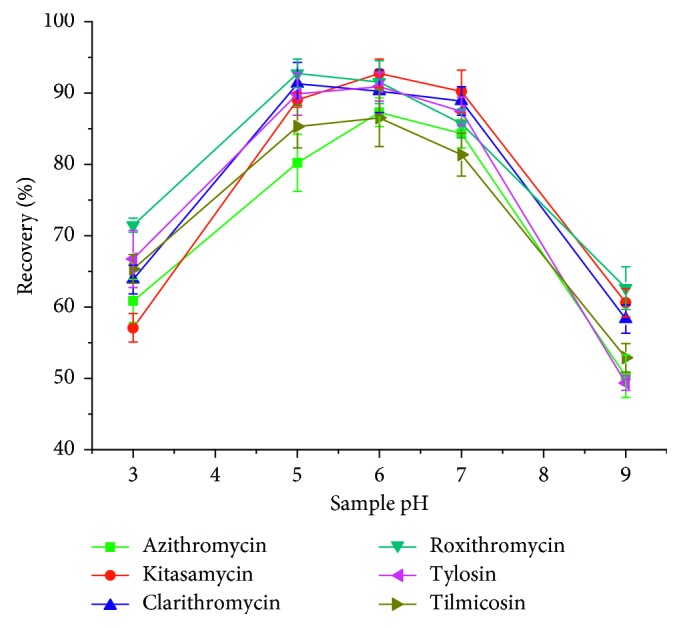
Effect of sample pH on macrolide antibiotics recovery.

The pH of sample solution plays an important role in SPE process because it could strongly affect the surface charge of the sorbent and the ionic or neutrality state of target analytes and further affected the extraction efficiency accordingly [34]. The effects of pH on the recoveries of MACs were investigated in the pH range of 3–9. From the results (Figure 9), the highest recovery was obtained when the pH was 6. This can be attributed to two reasons. On the one hand, too low pH may destroy the adsorption capacity of PAF-6 and lead to the low recoveries. On the other hand, most MACs exists in the form of ions under weakly alkaline conditions, which can significantly weaken the hydrogen bonding interaction between MACs and PAF-6, and leads to the low recoveries. Thus, pH 6 was optimized for the following experiments.

#### 3.3.6. Reusability of the PAF-6

**Figure 10:**
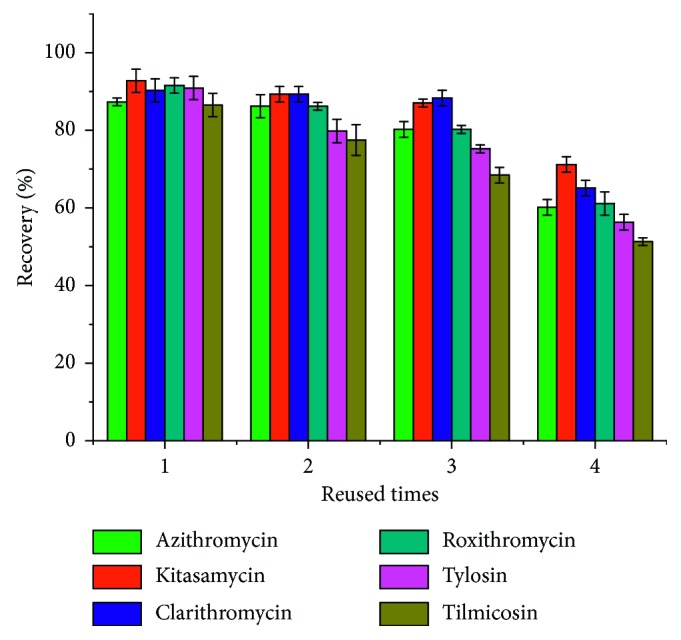
The average recovery after PAF-6 being consecutively regenerated.

In order to investigate the properties of the porous material PAF-6 sorbents, we examined the reusability of this material. The results indicated that, as shown in Figure 10, the recoveries of MACs only slightly reduced when it was used after three times. However, in order to make the experimental results more accurate, we used the self-made SPE column for two times in this work.

### 3.4. Method Validation

**Figure 11:**
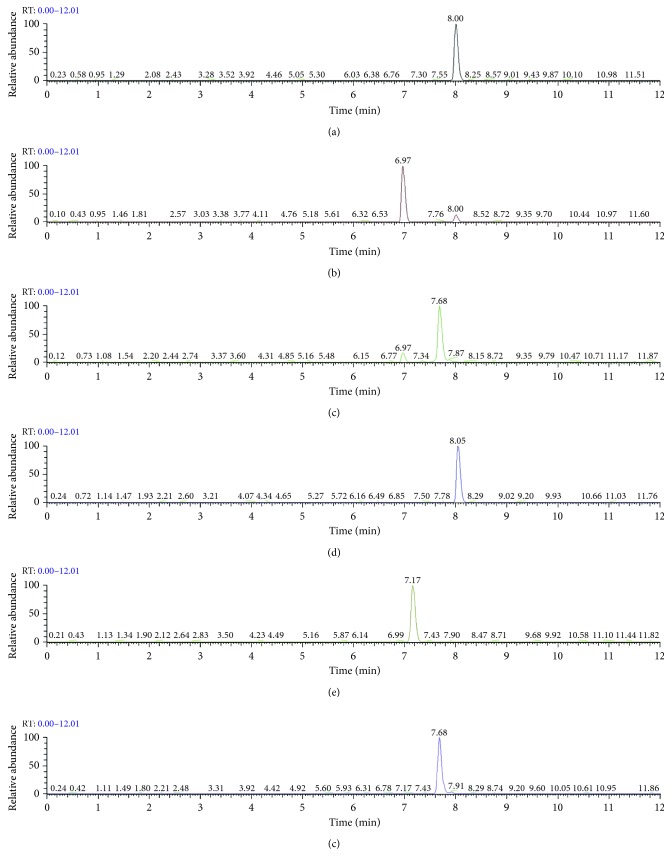
Liquid chromatography-tandem mass spectrometry chromatogram of extract of blank chicken sample spiked with six macrolide antibiotics equivalent to the limit of quantifications. (a) Clarithromycin. (b) Azithromycin. (c) Kitasamycin. (d) Roxithromycin. (e) Tilmicosin. (f) Tylosin.

To investigate the suitability and practicability of this method regarding determination of MACs in chicken samples, a series of parameters in experiment were validated. Under the above-optimized conditions, the method validation parameters are presented in Table 4. LC‐MS/MS chromatogram of the chicken sample spiked with MACs equivalent to the limit of quantifications (LOQs) is shown in Figure 11. The method showed good linearity over the concentration range from 2.5 *μ*g·kg^−1^ to 100 *μ*g·kg^−1^ for tylosin, tilmicosin, and kitasamycin, and 1 to 40 *μ*g·kg^−1^ for azithromycin, clarithromycin, and roxithromycin. The limit of detections (LODs) values ranged from 0.2 to 0.5 *μ*g·kg^−1^ based on a signal-to-noise (s/n) ratio of 3. The precision of the method was evaluated by analyzing the spiked products at three concentrations levels (as shown in Table 5), and every solution was measured in triplicate. As can be seen from Table 5, the average recoveries of the six MACs ranged from 82.1% to 101.4% with the relative standard deviations (RSDs) less than 11.1%. The intraday RSD was determined by analyzing on the same day six replicates and the interday RSD was evaluated doing three replicates in three difference days. From these results, the developed method was accurate and reproducible.

### 3.5. Application to Real Samples

To demonstrate the applicability of the method developed in this study, we applied the established SPE-LC-MS/MS method for the determination of residual contents of six MACs in chicken samples. Ten batches of chicken samples (provided by the Henan Province Bureau of animal husbandry) from different producing areas in Henan Province were detected. Tylosin was detected in two samples with the contents of 38.752 *μ*g·kg^−1^ and 79.211 *μ*g·kg^−1^, respectively. Azithromycin and tilmicosin were detected in one sample, and the contents were 27.336 *μ*g·kg^−1^ and 56.719 *μ*g·kg^−1^, respectively. The results of six MACs in chicken samples were determined and are shown in Table 6.

### 3.6. Comparison of the Proposed Method with Previously Reported Results

The performance of the developed extraction method was compared with some other reported methods for the analysis of macrolide antibiotics. As shown in Table 7, liquid extraction, pressurized liquid extraction, solid-phase extraction, and magnetic solid-phase extraction have been used for the determination of different antibiotics in meat samples or other food samples. In comparison with various methods (Table 7), the first four ways of sample preparation were simple and fast, which used liquid extraction and a dilution step prior to direct injection into the instrument, but there was a high risk of mass spectrometry source contamination due to injecting “dirty” extracts. Compared with the last three approaches using magnetic molecularly imprinted polymers or commercial SPE cartridges (Oasis HLB cartridge and Bond-Elut C18 SPE cartridge) [5, 24, 50], the developed method in this work exhibited lower LODs and higher recoveries.

In comparison, the developed extraction method was demonstrated to be a simple, sensitive, effective one for determination of macrolide antibiotics in the chicken sample. Therefore, the developed SPE procedure coupled with LC-MS/MS could become an alternative tool for analyzing the residues of MACs in food samples.

## 4. Conclusions

In this work, the PAF-6 was successfully applied to extract and purify MACs from the chicken samples. Compared with the traditional commercial SPE cartridge, PAF-6 SPE cartridge showed good adsorption capacity and reproducibility, which greatly saved the experimental cost. The developed LC-MS/MS based on PAF-6 SPE was reliable, sensitive, accuracy, and practical for the determination of MACs in the chicken samples. The method is a promising candidate for use in the food safety monitoring.

## Figures and Tables

**Table 1:** Operation parameters of macrolide antibiotics in selective reaction monitoring mode.

Compound	Formula	Precursor ion (*m*/*z*)	Product ion (*m*/*z*)	Collision energy (V)
Tylosin	C_46_H_77_NO_17_	916.583	174.000^*∗*^	36.444
			772.333	27.140
Tilmicosin	C_46_H_80_N_2_O_13_	869.591	696.333^*∗*^	39.680
			174.000	43.573
Azithromycin	C_38_H_72_N_2_O_12_	749.539	591.333^*∗*^	26.230
			573.347	32.146
Clarithromycin	C_38_H_69_NO_13_	748.491	158.000^*∗*^	26.230
			590.276	16.978
Roxithromycin	C_41_H_76_N_2_O_15_	837.570	679.333^*∗*^	18.798
			158.000	31.489
Kitasamycin	C_41_H_69_NO_13_	772.470	109.000^*∗*^	38.213
			558.222	23.702

^*∗*^Ions for quantitation; other ions are for confirmation.

**Table 2:** Adsorption energies (Δ*E*_*i*_) for different sites determined using the B3LYP method combined with the 6-31G basis.

PAF-6-analytes	Δ*E*_*i*_ (kcal/mol)
PAF-6-azithromycin	−23.47
PAF-6-clarithromycin	−16.21
PAF-6-roxithromycin	−12.13
PAF-6-tilmicosin	−5.58
PAF-6-tylosin	−21.25
PAF-6-kitasamycin	−3.45

**Table 3:** The liquid chromatography gradient elution method.

Time (min)	*A* (%)	*B* (%)
0	95	5
1.5	95	5
3	80	20
5	40	60
7	5	95
10	5	95
10.1	95	5
12	95	5

**Table 4:** Method validation parameters for determination of macrolide antibiotics.

Analyte	Linear equation	Correlation coefficient (*r*)	Limit of detection (*μ*g·kg^−1^)	Limit of quantification (*μ*g·kg^−1^)
Tylosin	*y* = 4.769*e*4*x* − 7.013*e*4	0.9942	0.5	1.8
Tilmicosin	*y* = 7.169*e*4*x* − 4.283*e*4	0.9982	0.5	1.8
Azithromycin	*y* = 2.081*e*5*x* + 5.737*e*4	0.9987	0.2	0.8
Clarithromycin	*y* = 3.931*e*5*x* − 9.809*e*4	1.0000	0.2	0.8
Roxithromycin	*y* = 1.599*e*5*x* − 5.39*e*4	0.9996	0.2	0.8
Kitasamycin	*y* = 1.957*e*5*x* − 3.47*e*4	0.9964	0.5	1.8

**Table 5:** Analytical results of macrolide antibiotics in samples.

Sample	Analyte	Amount spiked (*μ*g·kg^−1^)	Detection value (*μ*g·kg^−1^)	Recovery (%)	RSD (%)	
					Intraday	Interday
Chicken	Tylosin	2.5	2.54	101.4	3.7	7.9
		10	8.93	89.3	3.5	5.6
		50	45.61	91.2	6.7	9.6
	Tilmicosin	2.5	2.42	96.7	4.2	7.5
		10	8.59	85.9	2.5	4.6
		50	44.35	88.7	2.1	4.5
	Azithromycin	1	0.82	82.1	5.6	9.8
		5	4.45	88.9	2.3	6.3
		20	16.92	84.6	3.9	8.1
	Clarithromycin	1	0.97	97.0	3.4	5.2
		5	4.09	82.8	5.9	10.6
		20	18.08	90.4	1.7	3.5
	Roxithromycin	1	0.87	87.4	4.1	7.1
		5	0.50	100.1	3.8	8.2
		20	16.70	83.6	5.1	10.3
	Kitasamycin	2.5	2.31	92.3	3.5	6.9
		10	8.61	86.1	5.1	11.1
		50	43.90	87.8	2.7	7.1

**Table 6:** Results of the macrolide in real chicken samples (*n*=3).

Analyte	Chicken sample 1		Chicken sample 2		Chicken sample 3		Chicken sample 4		Chicken sample 5		Chicken sample 6		Chicken sample 7		Chicken sample 8		Chicken sample 9		Chicken sample 10	
	Found (*μ*g/kg)	RSD (%)	Found (*μ*g/kg)	RSD (%)	Found (*μ*g/kg)	RSD (%)	Found (*μ*g/kg)	RSD (%)	Found (*μ*g/kg)	RSD (%)	Found (*μ*g/kg)	RSD (%)	Found (*μ*g/kg)	RSD (%)	Found (*μ*g/kg)	RSD (%)	Found (*μ*g/kg)	RSD (%)	Found (*μ*g/kg)	RSD (%)
Tylosin	/	/	/	/	38.752	5.74	/	/	79.211	6.68	/	/	/	/	/	/	/	/	/	/
Clarithromycin	/	/	/	/	/	/	/	/	/	/	/	/	/	/	/	/	/	/	/	/
Azithromycin	/	/	27.336	3.56	/	/	/	/	/	/	/	/	/	/	/	/	/	/	/	/
Roxithromycin	/	/	/	/	/	/	/	/	/	/	/	/	/	/	/	/	/	/	/	/
Kitasamycin	/	/	/	/	/	/	/	/	/	/	/	/	/	/	/	/	/	/	/	/
Tilmicosin	/	/	56.719	4.83	/	/	/	/	/	/	/	/	/	/	/	/	/	/	/	/

**Table 7:** Comparison of different analytical methods for the determination of macrolide antibiotics in food samples.

Sample	Method of sample preparation	Sorbent	Instrument technique	Limit of detection (*μ*g·kg^−1^)	Reference
Meat	Accelerated solvent extraction	—	LC-MS/MS	<0.61	[1]
Fish	Extracted with trichloroacetic acid	—	LC-MS/MS	0.4–74.24	[47]
Chicken	Two-step extraction	—	LC-MS/MS	0.20–1	[48]
Meat and fish	Pressurized liquid extraction (PLE)	—	LC-(ESI)MS	20–51	[49]
Foodstuff	Magnetic solid-phase extraction (MSPE)	Magnetic molecularly imprinted polymers (MMIPs)	HPLC-UV^a^	15–200	[5]
Royal jelly	Liquid-phase extraction and SPE	Oasis HLB cartridges	LC-MS/MS	0.4–2	[50]
Tissues	SPE	Bond-Elut C18 SPE cartridge	LC-MS/MS	0.5	[24]
Chicken	Solid-phase extraction (SPE)	PAF-6	LC-MS/MS	0.20–0.50	This work

^a^HPLC-UV: high-performance liquid chromatography-ultraviolet.

## Data Availability

The data used to support the findings of this study are included within the article.
